# Rehabilitation of a Child with Denari Prosthesis after Dental Avulsion

**DOI:** 10.1155/2022/7473642

**Published:** 2022-06-24

**Authors:** César Sebastián Arcos-López, Juan Marcos Parise-Vasco, Ana Armas-Vega, Yecenia Carrillo-Azuero

**Affiliations:** ^1^Universidad UTE, Facultad Ciencias de la Salud “Eugenio Espejo”, Quito, Ecuador; ^2^Universidad UTE, Facultad Ciencias de la Salud “Eugenio Espejo”, Centro de Investigación en Salud Pública y Epidemiología Clínica (CISPEC), Quito, Ecuador; ^3^Universidad UTE, Facultad Ciencias de la Salud “Eugenio Espejo”, Centro de Investigación en Salud Oral (CISO), Quito, Ecuador

## Abstract

Prosthetic rehabilitation in cases of premature loss of temporary teeth always is a procedure that must be carried out in a planned and integral manner. The avoidance of opposing tooth extrusion, loss of horizontal space, and acquisition of bad oral habits are fundamental to not trigger alterations in the child's occlusal development and psychological development. The purpose of this document is to report the clinical case of a 4-year-old patient with avulsion of the anterior primary teeth secondary to severe automobile trauma. In this case, we describe the use of a Denari-type fixed partial prosthesis. This prosthesis can recover the function and aesthetics of the superior anterior sector of the oral cavity, thus preventing lingual interposition. The treatment with the Denari prosthesis allowed satisfactory results relating to the transverse growth of the maxillary bone in this case report.

## 1. Introduction

Dentoalveolar trauma represents a public health problem in children and adolescents [1], with mild consequences such as soft tissue laceration, up to severe consequences such as dental avulsion [2]. Dental issues could trigger functional and even psychological disorders that have repercussions on the patient's quality of life and their family [3, 4]. In the face of this type of trauma in primary dentition, the objectives should include pain management and prevention of damage to the permanent successor tooth germ [5]. Medium- and long-term follow-ups are required to identify late sequelae associated with eruption disorders and alterations in morphogenesis [6, 7], secondary to energy transmission from the impact to the tooth germ [8].

Lateral dislocations, intrusions, and dental avulsions are found in the group of traumatic injuries associated with severe complications [9]. Dental avulsions range between 7% and 13% of these injuries [10]. This trauma is characterized by the complete displacement of a tooth from its alveolar support and the premature loss of it [11], with repercussions on the growth of maxillary bones [12]. In this context, the literature refers to different types of fixed appliances, to temporarily replace these lost deciduous teeth, until the eruption of permanent teeth [10, 11].

The Denari prosthesis is a fixed prosthetic device, not very well known, indicated for the premature loss of one or two anterior teeth with the purpose of restoring the aesthetic of the upper front teeth, restoring oral function, and interfering with the tongue thrusting habits. It is necessary to carry out the treatment based on a Denari fixed partial prosthesis. This includes a fixed restoration to replace teeth 5.1 and 6.1 with acrylic bridges, a central stem, and anchorage to teeth 5.2 and 6.2. The central stem is not rigid; it is connected to a tube present in one of the protuberances with the function of activating the prosthesis, allowing a small distance between the retainer and the protuberance through the movement of the stem. This allows monitoring of the lateral growth of the premaxilia [13]. In addition to its aesthetics, it controls the inappropriate lingual position avoiding the development of harmful speech without interfering with the appropriate growth pattern [14].

We aim to report the clinical case of a 4-year-old patient, who presented an avulsion of the anterior primary teeth and received prosthetic treatment using a Denari-type fixed partial prosthesis.

## 2. Case Description

A four-year-old male patient came to the university clinic of the UTE University, Quito, Ecuador, in the company of his mother, who reported that a couple of months ago, the minor suffered a car accident, in which the boy lost his upper incisor teeth. During the intraoral clinical examination, the absence of teeth 5.1 and 6.1 was confirmed. Additionally, an incisal fracture of tooth 8.1 and caries in tooth 8.5 were observed.

The clinical and radiographic findings in the panoramic and periapical records, together with the analysis of the models obtained after the impression of the dental arches, were discussed with the parents. We discussed the treatment plan to be carried out, with its possible complications and benefits. The signature on the informed consent documents by the parents allowed us to start with oral physiotherapy and instruction of good oral hygiene habits. We highlighted to the minor and his parents the importance of a correct brushing technique.

**Figure 1:**
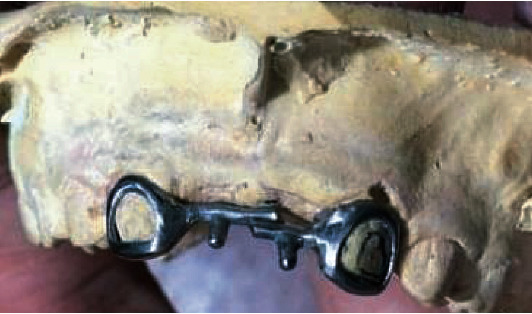
Design of a bar-tube system of the Denari prosthesis.

Rehabilitation procedures included removal of the carious lesions and adhesive restoration with an adhesive resin cement (RelyX™ Ultimate, 3M) on teeth 8.5 and 8.1. The cast models of the patient's mouth, together with a bite registration, were sent to the dental technician along with the design of a bar-tube system (Figure 1) [13]. The system constitutes a metal skeleton for the Denari fixed prosthesis, made of an alloy of nickel, molybdenum, chromium, and beryllium combined with the placement of artificial teeth of color A1 resin, seeking to replace teeth 5.1 and 6.1.

**Figure 2:**
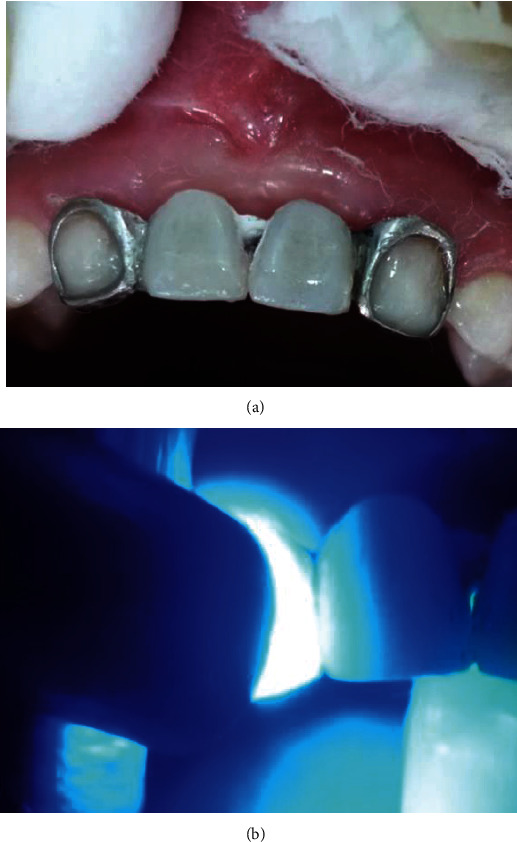
Rehabilitation procedures. (a) Cementation of device. (b) Photopolymerization.

After rigorous adaptation tests, the device was placed in the mouth of the patient. Previous to its application, the teeth adjacent to the edentulous space (5.2 and 6.2) were cleaned with pumice and 0.12% chlorhexidine. It was performed on teeth 5.2 and 6.2; the enamel of these teeth was conditioned with 37% orthophosphoric acid (3M-ESPE) for 15 seconds, followed by placement of the adhesive system (Scotchbond, 3M) with its subsequent photopolymerization. Simultaneously, the preparation of the prosthetic element was carried out with the placement of silane (Monobond Plus, Ivoclar Vivadent) for 60 seconds, followed by drying and impregnation of the adhesive system, with its subsequent photopolymerization, in the retainers of the metal skeleton of the Denari prosthesis (Figure 2).

Dual-polymerized resin cement was used in the cementation of the previously prepared retainers on teeth 5.2 and 6.2. Following the manufacturer's recommendations, it was light-cured for 4 seconds, the excess material was eliminated, and then, the light-curing process was concluded, performing the elimination of interference and occlusal control. We sought to improve the aesthetics, so that the metallic structures of the prosthesis seated on teeth 5.2 and 6.2 were covered with composite resin (Z350, 3M), color A1. The appropriate polymerization was performed as well as the corresponding finishing and polishing procedures.

**Figure 3:**
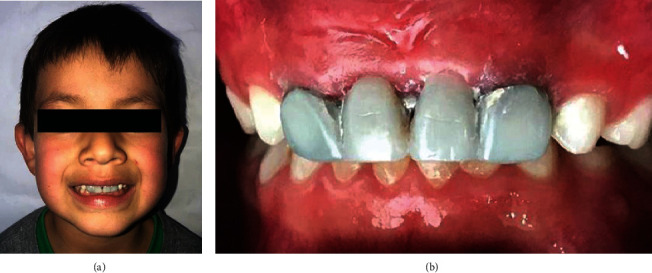
Three-month control appointment.

**Figure 4:**
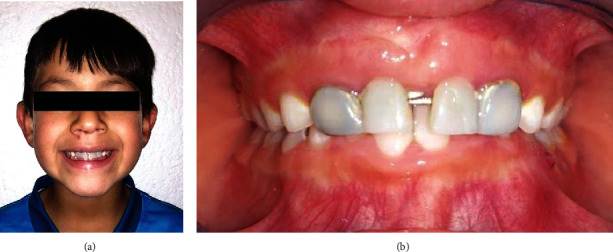
One-year control appointment. (b) clearly shows an increase in the interincisal spacing that was the resultant of transverse maxillary growth, which is the rationale of using this appliance type.

Recommendations were provided to the parents for maintenance, care, and control appointments every three months for one year to monitor and evaluate the transverse growth of the maxilla (Figure 3). On a year control, a passive transversal growth was observed with an increase of the interincisor space of approximately 3.5 mm. It is important to mention that the prosthesis has aesthetic purposes and acts passively, so it does not exert any action over dental or osseous tissues. Any change in the occlusion is only given by the normal maxillofacial growth of the patient (Figure 4).

## 3. Ethical Considerations

The present case report was performed after the patient's legal guardian signed the informed consent form and agreed with the treatment plan. In addition, the legal guardian approved the use of the patient's clinical information and photographs for presenting them anonymously for publication in a scientific journal.

## 4. Discussion

The use of the Denari prosthesis as a replacement for teeth 5.1 and 5.2 allowed achieving the objectives set in the treatment plan, concurring with the literature that reports its use after the loss of upper anterior teeth [2]. Faced with the premature loss of temporary anterior teeth, maintaining the space is an important goal to avoid harmful habits [15]. It is also necessary for the normal development of the functions of the stomatognathic system and to avoid involuntary exploration of the edentulous space with oral structures such as the tongue or external elements (e.g., thumb). This involuntary exploration can cause maxillary protrusion or protractile tongue, triggering swallowing patterns with alteration of muscular activity and malocclusion [5].

Although the literature refers to a series of fixed devices to be used in similar cases to the one above drafted [10], such as fixed prostheses with orthodontic bands, fixed prostheses with precision attachments, and the Nance button, among others [11], with advantages associated with a minimum dental preparation, easy repair, and absence of the possibility of damaging periodontal tissues [12], these require a simultaneous modification with the growth and development of the premaxilla in a transverse direction, which triggers a constant replacement of these devices [16]. The prosthetic design, based on the tube-bar system, which characterizes this prosthesis, in addition to being readily accepted by the patient, guarantees minimal repercussion of soft and hard tissues, thus achieving adequate and correct occlusion, without interfering with growth maxillary transverse [13, 17]. This outcome was evidenced in our patient, in whom an increase in the interincisal spacing was observed as a result of transverse maxillary growth, which is the rationale of using this appliance type.

The planning of the prosthetic treatment of a pediatric patient requires the analysis of the patient's age, capacity for cooperation, or behavior, but above all, the stage of language development [18], chewing function, and the presence of harmful habits. These elements were also determinant factors in the decision to use this device. One of the strengths in the present case is the preservation of the tissue in which it is placed and the maintenance of the device until the eruption of the successor tooth [9], with its adjustment related to normal maxillary growth. There are advantages that make it an excellent alternative to a removable device [16] and even a fixed device in young patients [2].

For a pediatric patient, it is imminent that the adaptation of any intraoral device is a cumbersome process. However, the friendly and conservative design of the Denari prosthesis ensures its use, especially when the age and cooperation of the patient are interfering. In this context, a disadvantage found in this case is the difficulty in the cleaning process of the device by the patient, hence the need for conditioning, motivation, and training of their parents, who are required to carry out daily oral physiotherapy processes. Another limitation was the esthetic compromise; however, a series of procedures were employed such as microsandblasting of the metal material to achieve adhesion of the resin to the metal and hide to some extent the metal structure.

Although the present report indicates the execution of the procedure, the restricted literature regarding the Denari prosthesis constitutes a significant limitation. We suggest carrying out a randomized clinical trial to analyze the outcomes of this type of prosthesis.

## Data Availability

No data were used to support this study.
